# IDAAPM: integrated database of ADMET and adverse effects of predictive modeling based on FDA approved drug data

**DOI:** 10.1186/s13321-016-0141-7

**Published:** 2016-06-14

**Authors:** Ashenafi Legehar, Henri Xhaard, Leo Ghemtio

**Affiliations:** Centre for Drug Research, Division of Pharmaceutical Biosciences, Faculty of Pharmacy, University of Helsinki, Viikinkaari 5E, 00790 Helsinki, Finland; Division of Pharmaceutical Chemistry and Technology, Faculty of Pharmacy, University of Helsinki, P.O. Box 56, 00014 Helsinki, Finland

**Keywords:** FDA approved drugs, ADMET, Adverse effects, Targets, Database, Predictive modeling, Drug-target database, Data analysis

## Abstract

**Background:**

The disposition of a pharmaceutical compound within an organism, i.e. its Absorption, Distribution, Metabolism, Excretion, Toxicity (ADMET) properties and adverse effects, critically affects late stage failure of drug candidates and has led to the withdrawal of approved drugs. Computational methods are effective approaches to reduce the number of safety issues by analyzing possible links between chemical structures and ADMET or adverse effects, but this is limited by the size, quality, and heterogeneity of the data available from individual sources. Thus, large, clean and integrated databases of approved drug data, associated with fast and efficient predictive tools are desirable early in the drug discovery process.

**Description:**

We have built a relational database (IDAAPM) to integrate available approved drug data such as drug approval information, ADMET and adverse effects, chemical structures and molecular descriptors, targets, bioactivity and related references. The database has been coupled with a searchable web interface and modern data analytics platform (KNIME) to allow data access, data transformation, initial analysis and further predictive modeling. Data were extracted from FDA resources and supplemented from other publicly available databases. Currently, the database contains information regarding about 19,226 FDA approval applications for 31,815 products (small molecules and biologics) with their approval history, 2505 active ingredients, together with as many ADMET properties, 1629 molecular structures, 2.5 million adverse effects and 36,963 experimental drug-target bioactivity data.

**Conclusion:**

IDAAPM is a unique resource that, in a single relational database, provides detailed information on FDA approved drugs including their ADMET properties and adverse effects, the corresponding targets with bioactivity data, coupled with a data analytics platform. It can be used to perform basic to complex drug-target ADMET or adverse effects analysis and predictive modeling. IDAAPM is freely accessible at http://idaapm.helsinki.fi and can be exploited through a KNIME workflow connected to the database.

**Graphical abstract:**
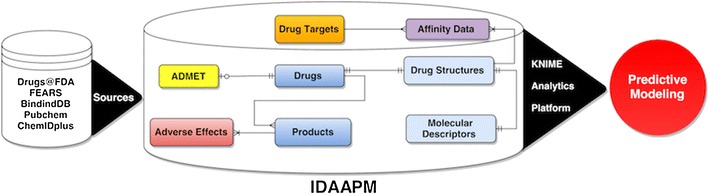
FDA approved drug data integration for predictive modeling

**Electronic supplementary material:**

The online version of this article (doi:10.1186/s13321-016-0141-7) contains supplementary material, which is available to authorized users.

## Background

Absorption, Distribution, Metabolism, Excretion, Toxicity (ADMET) properties and adverse effects are considered to be responsible for the late stage failure of many promising compounds as well as for the withdrawal of approved drug molecules. Despite considerable efforts to improve the pharmacokinetic profiles of small-molecule drug candidates, overall attrition rates remain high mainly due to efficacy, safety issues, and selection of inappropriate drug targets [1–3].

Computational prediction of ADMET properties and adverse effects is an effective method to minimize the risk of late-stage attrition and reduce the number of safety issues. This method is now well established as a reliable and cost-effective approach to assist the drug discovery process. Computational models are used to focus medicinal chemistry efforts into the suitable chemical space; to connect, use and extend experimental data; to minimize the number of compounds to be synthesized; as well as to obtain a favorable biochemical and/or physicochemical profile [4–12]. For example, multiple studies have explored the benefit of controlling the size, lipophilicity and polarity properties of compounds in terms of reduced likelihood of attrition [4–8, 13–15].

Currently, a large amount of data is made available by the pharmaceutical industry and academic research groups that can be used for computational predictions. These data have been deposited in databases, among which the most well-established and freely accessible are DrugBank [16], ChEMBL [17], BindingDB [18], PubChem [19], PDB [20], PDBbind [21], GtoPdb [22], Therapeutic Target Database [23] and ChemIDPlus [24]. DrugBank integrates detailed chemical, pharmacological and pharmaceutical drug data with target information. ChEMBL contains the chemical structures and bioactivity data of compounds with drug-like properties. BindingDB and PDBbind provides ligand–target interaction experimental affinity data that are mainly collected from scientific literature and other auxiliary databases such as ChEMBL. GtoPdb (previously IUPHAR-DB) contains ligand information and special sections for receptors, ion channels, kinases and transporters. Therapeutic Target Database (TTD) provides information about the known and explored therapeutic protein and nucleic acid targets such as disease, pathway and drugs connected to each of these targets. ChemIDPlus focus more on molecular and structural information of compounds. The US food and drug administration (FDA) has two publicly available databases; Drugs@FDA [25] which is the main resource that provides FDA-approved drug information, and FAERS (FDA adverse event reporting system), which contains a collection of reported post-marketed adverse effects [26, 27].

Focused and integrated databases with predictive ADMET and adverse effects models have been developed to exploit this data. UCSF-FDA TransPortal [28] focus on interaction of drug molecules with transporters, leading to drug–drug interactions. For example, Sedykh et al. collected and published a large collection of transporter interaction data for small molecules focused on major human intestinal transporters with the aim of building predictive models [29]. Moda et al. [30] developed a database, PK/DB associated with five in silico ADME models to predict human intestinal absorption, human oral bioavailability, plasma protein binding, blood-brain barrier and water solubility. Kuhn et al. have developed a database called SIDER [31], which contains adverse effects of drugs and their frequency, however, unlike FAERS which is a reporting system, SIDER extracts the causal relation from the drug label. In another recent study, Cheng and coworkers developed a meta-database of drug adverse effects, MetaADEDB, which includes the SIDER data [32].

Although these resources possess an already large amount of ADMET or adverse effects data, they all have different aims and contents. Consequently, they do not integrate additional related data (e.g. approval application, affinity data, molecular descriptors, data references, adverse effects) and metadata required for in-depth predictive modeling analyses. It therefore requires tedious data preparation and cleaning to exploit these data for predictive modeling of ADMET properties or adverse effects. Therefore, a unified approved drugs information database, combined with fast and efficient predictive tools is desirable.

Here we describe IDAAPM, a publicly available database of FDA approved drugs, which have been developed as a useful resource for computational analyses and modeling. IDAAPM aims to bridge the gap by providing, in a single resource, integrated detailed information on approved drugs (small molecules and biologics) such as FDA application data, structures, molecular descriptors, ADMET properties and adverse effects, target and also related bioactivity data that are often missing in other comparable databases. This resource can be used to analyze relevant information across studies based on compound similarities and the chemical space associated with drug molecules. It would become possible to easily infer relationships among physicochemical properties and ADMET properties and adverse effects based on how new compounds overlap with the space of approved drugs and their targets for new compounds. IDAAPM is coupled with KNIME [33], a modern data analytics platform to allow data access, data transformation, initial analysis, visualization and predictive modeling. KNIME platform implements a modular approach to workflow management and allows the flexibility to incorporate different tools and also create specialized workflows that are easy to use for automation.

## Construction and content

### Data source

Approved drugs (small molecule and biologics) application information was collected from the FDA resource Drug@FDA. For each drug entry, the standard drug information was collected, including trade names, administration routes, dosage, and approval data. Similarly, adverse effect reports for each drug were taken from FAERS, among them; patient data (demographics and administrative) and drug related data (name, indication, dosage, drug name, route of administration, frequency). Adverse effects are reported using the “preferred terms” (PT) of the medical dictionary for regulatory activities (MedDRA) [34]. MedDRA has a hierarchical structure with five levels: system organ class (SOC), high level group, high level, PT and lowest level. FAERS data are entered in the system by health care professionals and consumers, which can lead to errors and non-normalized data, thus forming substantial barriers for data integration for the purpose of data analysis. In IDAAPM, we have linked the PT to their corresponding 26 SOC to solve typographical errors and the PT difference between MedDRA versions. This produces data ready for mining that are clean, normalized and aggregated into fewer classes.

The two dimensional structure of the approved drugs, molecular descriptors and ADMET properties and adverse effects were collected from DrugBank and cross checked with PubChem, and ChemIDplus. Binding affinities and drug target data were collected from BindingDB. Target information contains target name, target source organism and drugs binding affinity data (such as K_i_, IC_50_ and K_d_) with reference to the original publication. The chemical structures are stored in the database using the IUPAC international chemical identifier (InChi key and InChi code) and SMILES (simplified molecular-input line-entry system). Drug structures were checked for potential problems (incorrect structures, salts) and then normalized in the database using their InChi key to avoid duplicate entries. For each compound, external reference to DrugBank, PubChem, IUPHAR and ChEBI were added in order to facilitate cross-linking and cross checking. The overall flowchart depicting the different steps of the database construction with sources, contents and processes is presented in Additional file 1.

### Database design

**Fig. 1:**
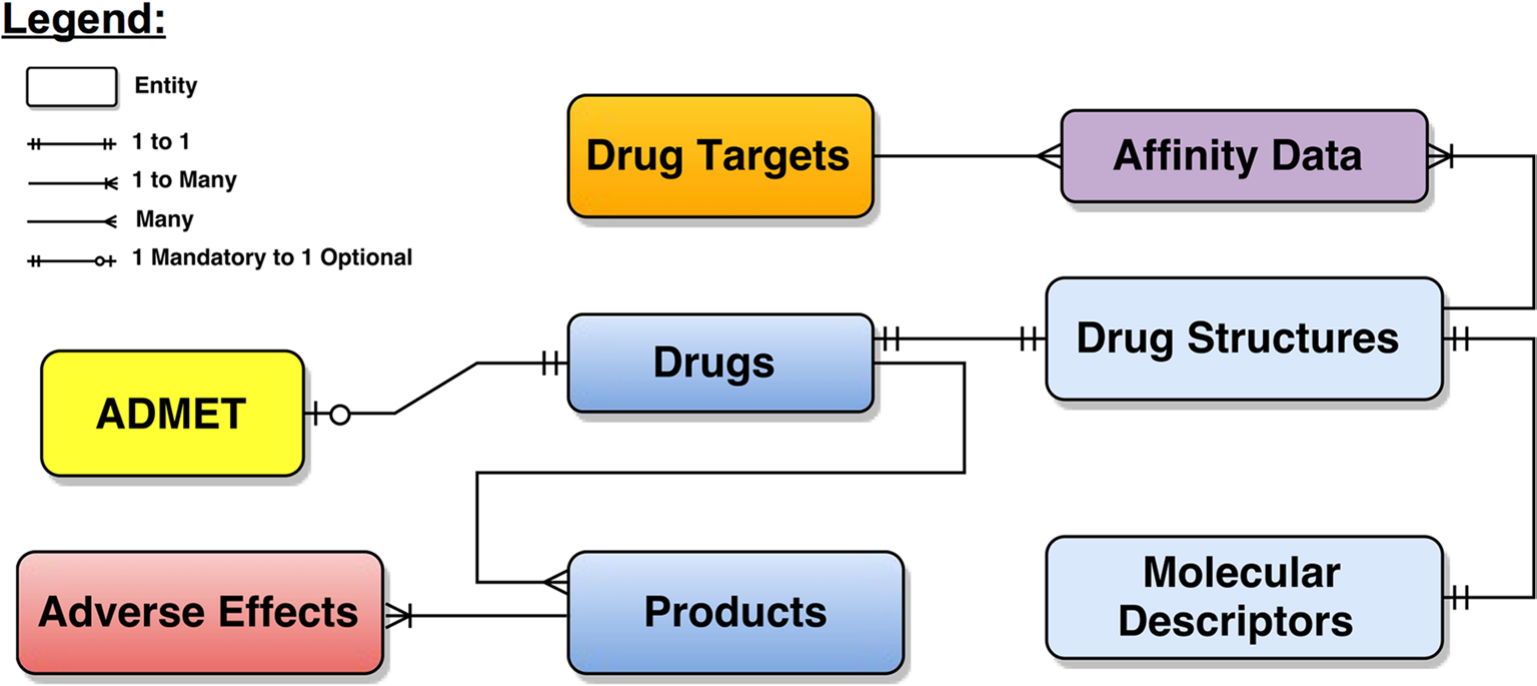
Simplified entity-relational model of IDAAPM. Eight entities or tables (main group of data) are shown linked by different types of association (see *legend*) to characterize how they are related to one another

IDAAPM is designed as an object-relational database, which is implemented in the Postgresql database server. A summarized model of the relational database structure is shown in Fig. 1. The overall detailed model and documentation are available in Additional files 2 and 3. The IDAAPM model is centered on products, adverse effects, drug structures, and targets tables. The first step of designing IDAAPM was to link approved drug information from FDA to chemical structures, targets and corresponding binding affinities. Then, for each drug molecule, molecular descriptors, ADMET and adverse effects data were extracted and filled. Identifying entities (main groups of information) and how they are related together were the most important step in the IDAAPM design. The last step was to normalize the database model, which allowed us to remove redundant information. This allows the database model to be flexible and reliable during maintenance, as well as to propagate to the entire database each update.

### Data preparation and integration

Data in IDAAPM was extracted from the resources listed above and supplemented with journal references when available (Additional file 1). First, all FDA approved drugs data were downloaded from Drug@FDA database in text file. Second, a .sql script (Additional file 4) was developed to extract and insert them in IDAAPM. The FAERS database was downloaded entirely into an .xml file, then an R script (Additional file 5) was developed to extract the adverse effects data of all approved drugs. Following this, a .sql script (Additional file 6) was written to insert the data into the IDAAPM database. Next, approved drugs data were downloaded from DrugBank into an .xml file, extracted with an R script (Additional file 7) and inserted into IDAAPM using a .sql script (Additional file 8). Then, PubChem and ChemIDplus were used to manually extract about 50 compound structures and molecular properties missing from DrugBank data. After that, drug-targets bioactivity data were downloaded from BindingDB as a .tsv file. A .sql script (Additional file 9) was developed to extract and insert the corresponding chemical structures, molecular descriptors, ADMET property information, drug target information and binding affinity as well as available literature references into IDAAPM. The SMILES and InChI code were cross-compared to those reported in PubChem and ChEMBL as an additional means to check for errors. Moreover, links to popular databases were also captured, if available. The curating process involved reading scientific articles (abstracts and full texts) then checking data to ensure that the correct drug names and affinity data had been assigned, followed by then manually cross checking the accuracy of data between different sources. A KNIME workflow module was developed to cross check the drug-target bioactivity data from PubChem and CheMBL. Special attention was given to the quality of chemical structures and bioactivity data. Finally, manual checking was performed on each entry as part of a continuous process. The binding affinity data unit of measurement are standardized preferred units of measurements for a given activity type. For instance, K_i_, IC_50_, K_d_ EC_50_ are recorded as nM, rather than µM or mM. This enables the user to easily compare data across different assays. We ensure that all bioactivity data in the database has been referenced. If a user finds a specific compound to be useful, they may follow the links to view literature available on that compound. Protein targets are further classified as receptor, enzyme, transporter, channel, kinase and others. This also allows data to be queried at a higher level.

### Current contents

**Fig. 2:**
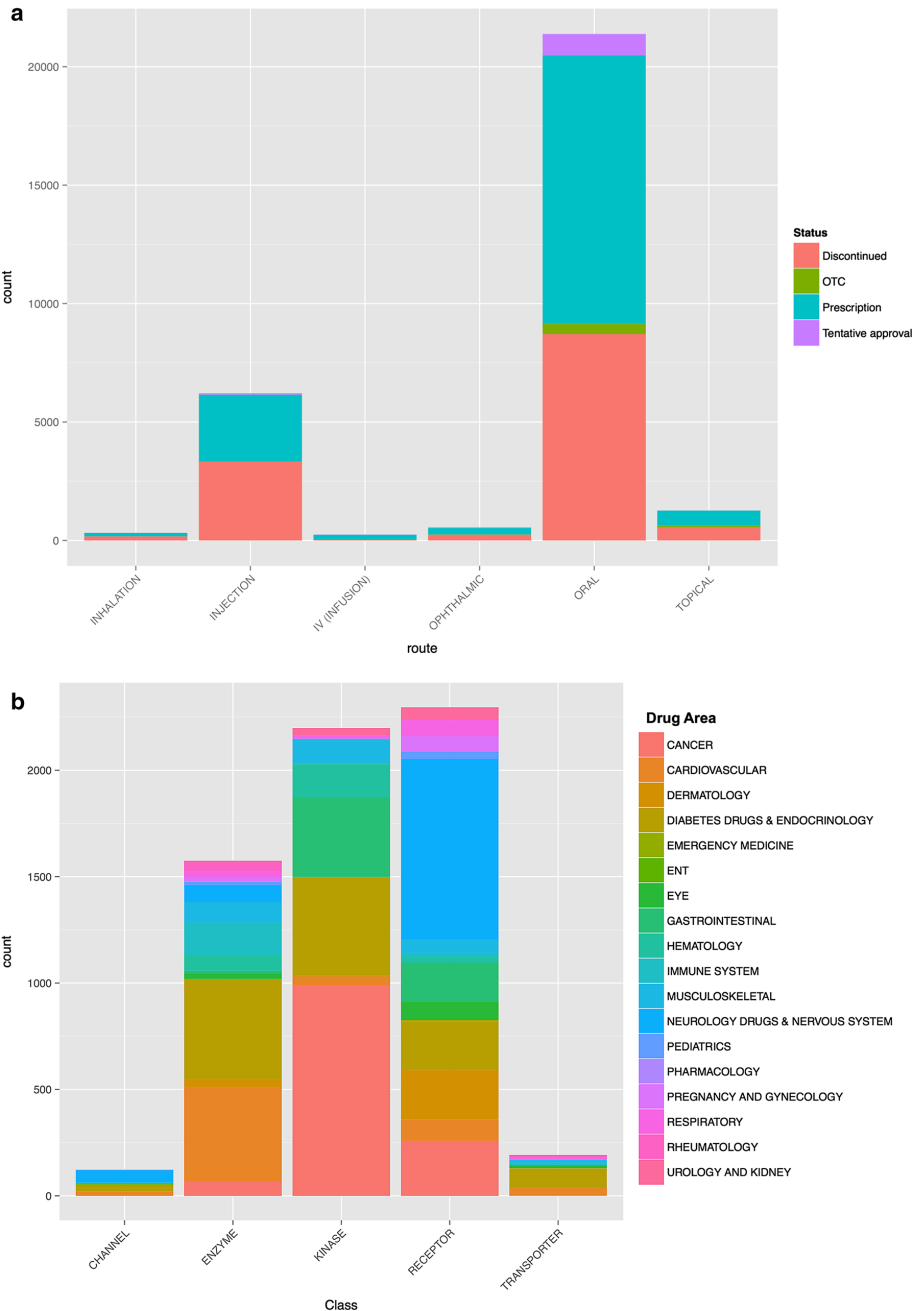
**a** Composition of IDAAPM drugs by mode of administration. Each *histogram bar* represents the amount of drugs present in IDAAPM for a selected mode of administration and is *colored* by commercial status of the drug. **b** Target distribution in IDAAPM by protein family. Each *histogram bar* represents the amount of target present in IDAAPM for the five most populated protein classes and is *colored* by their corresponding drug area

**Fig. 3:**
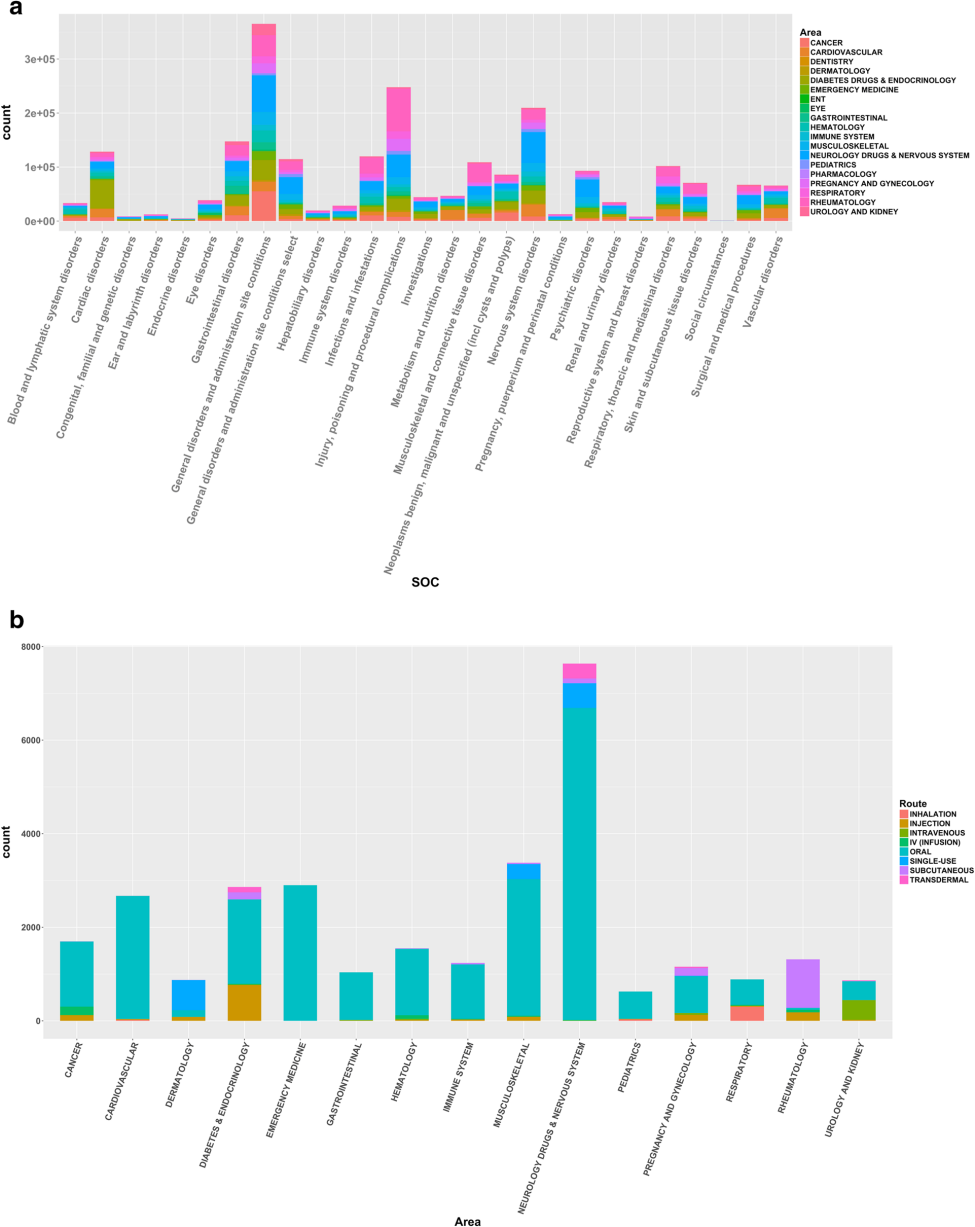
**a** Frequency of adverse effects. For the 26 adverse effects SOC MedDRA terms, each *histogram bar* corresponds to the amount of the selected adverse effect in IDAAPM and is *colored* by their corresponding drug area. **b** Systemic drugs distribution with ocular adverse effects. Adverse effects reported have relative frequency >0.1, each *histogram bar* corresponds to the primary area of systemic drugs with ocular adverse effect and is *colored* by their route of administration

A summary of the database content is shown in Table 1 (Additional file 10), which present the statistic of current IDAAPM database and their sources. IDAAPM is categorized into four major groups: (1) Approved drugs information, structure and physico-chemical properties; (2) ADMET properties; (3) Adverse effects; (4) Target and affinity data. The targets are defined by their Uniprot information (identification number and name) as well as the 3 letters PDB code when available. About 1629 FDA approved drug structures were collected, approximatively the amount present in most popular chemical databases (PubChem, DrugBank, GtoPdb and ChEBI). Adverse effects are the most populated at about 97 % (2.5 million) of the overall database, then the remaining data are drugs-targets affinity and FDA approved drug applications, as well as their related structural and physicochemical data. Figure 2a shows the composition of the database by mode of administration and drugs status information. There are a total of 20 prescription drug areas covered by the database, such as gastrointestinal, cardiovascular, diabetes and endocrinology, immune system, hematology, eye, cancer and so on. These prescription drug area classifications are collected from FDA definitions of a new drug approval or a new molecular entity. Figure 2b shows the distribution of the main target classes and drugs by therapeutic area. Adverse effects were grouped in 26 main SOC MedDRA terms; Fig. 3a shows their frequency and drugs by therapeutic areas.

**Table 1:** Summary of IDAAPM content

FDA applications	Products	Active ingredients	Structures	Drug areas	Target classes	Adeverse effects	Targets	Drug–targets interactions
14,260 Generics 4849 New drugs 117 Biologics Total: 19,226	31,815	2505	1629	20	6	2,472,329	3382	36,963

### KNIME workflows

**Fig. 4:**
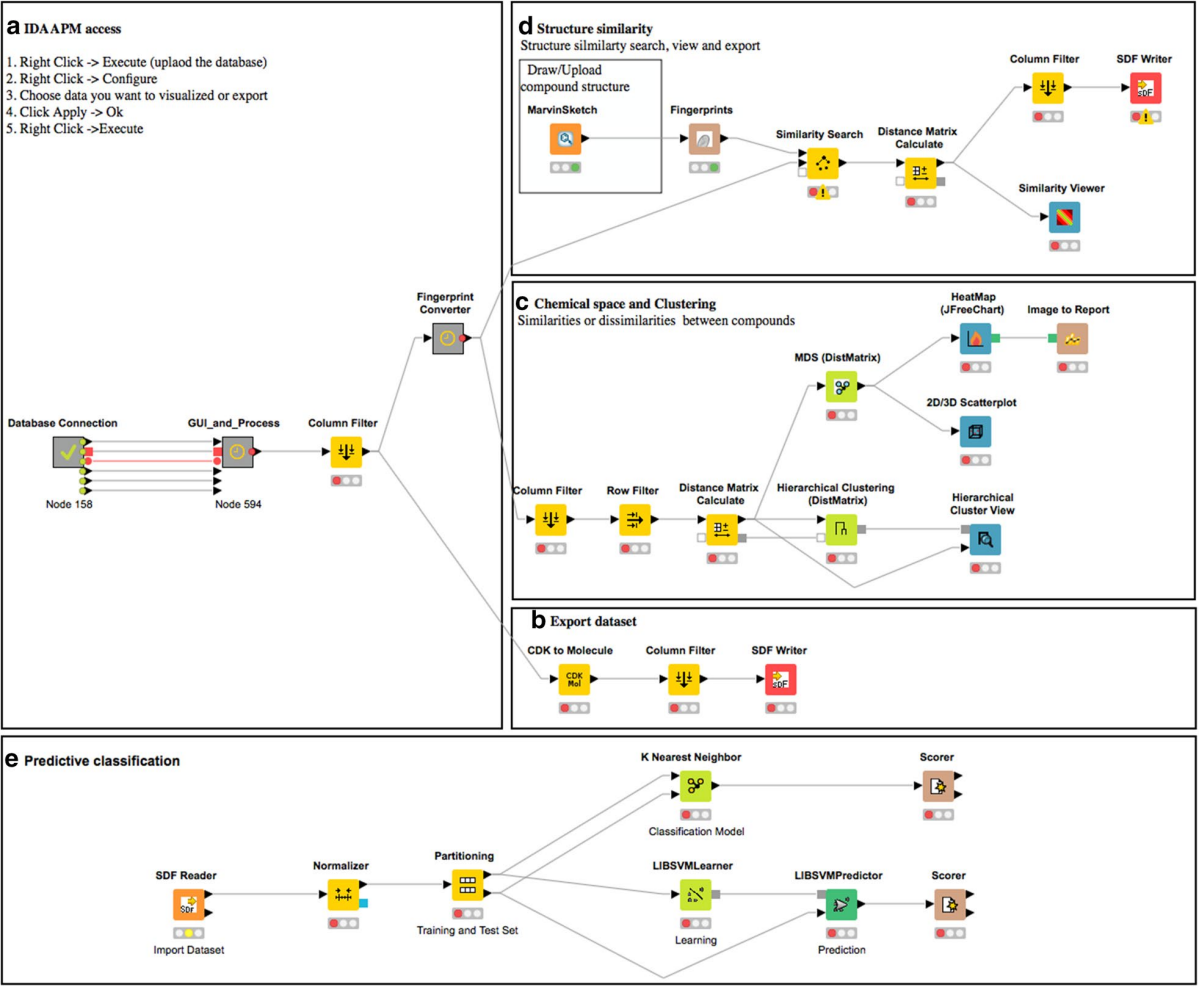
IDAAPM utility examples. Examples of KNIME workflows to access IDAAPM (**a**), export data (**b**), make preliminary analysis (**c**, **d**) and build predictive classification models (**e**)

KNIME is one of the most popular open-source programs in the field of chemoinformatics and bioinformatics. The KNIME analytics platform integrates tools for data preprocessing and cleaning, analysis, and modeling. Moreover, it contains data mining modules (Matlab, Weka, R) as well as interactive view environments and additional plugins allowing computational chemistry to be run. The platform offers access to a vast library of statistical routines and numerous libraries created by the scientific community and commercial software vendors. KNIME workflows were developed to allow access and query IDAAPM (Fig. 4a). These workflows include a series of constructive extraction, analysis, visualizing and computational steps (Fig. 4b–d). All the needed libraries have been integrated into the KNIME environment for that purpose.

## Utility

### Database access

IDAAPM is freely accessible via an online graphical user interface at (http://idaapm.helsinki.fi). The database browser was organized using a flask framework, java script, cascading style sheet and Jinja2 applications (Additional file 11A). The IDAAPM server was built with gunicorn, nginx and PostgreSQL 9.3.12, installed on Ubuntu 14.04.4. The user interface allows users to search the database by drug area, route of administration, target class, small molecules or biologics. Search with one of the above criteria allows retrieval of all matching drugs with their approval information, molecular descriptors, 2D structures, targets (Additional file 11B) and related bioactivity as well as adverse effect frequency and links to the literature references from which the data originated (Additional file 11C). From the download page, users can download the entire database as TSV/CSV format or a specific subset (small molecules/biologics) as well as molecular structures (SMILES format). A database dump is also available for download enabling users to install their own local copy of the database.

Finally, and most importantly, IDAAPM can be exploited through KNIME workflows (can be downloded from http://idaapm.helsinki.fi) connected to the database. The KNIME access (Fig. 4a) workflow enables the database to be queried by drug name, active ingredient, FDA application number, SMILES, sub-structural and structural similarity search (Additional file 12). The user has the possibility to build complex queries to search the database. IDAAPM provides different levels of ADMET properties and adverse effects search. At the first level, the search can be performed by (1) choosing to view all drug information, (2) searching a specific compound using drug name or active ingredient or (3) compound searching by drug area. At the second level, searching can be performed using drug area, route of administration and drug target class to further filter the results. More specific searches can also be performed using FDA application number, brand name, and active ingredient or drug area. Similarly, results obtained can be later exported as .sdf files (Fig. 4b). Compound searching can be filtered by drug area as well, such as gastrointestinal, Cardiovascular, Diabetes Drugs and Endocrinology, Eye, Hematology and so on. At the lowest level, the search criteria set up in the two previous levels can be completed with more additional data, using the check boxes in the last section (Additional file 12).

### Database usage

Clean, structured and high quality data stored in a relational database resource can facilitate computational predictive modeling, thus allowing for new information to be inferred by computational analyses. Data mining using resources like IDAAPM can be employed to understand, for example, target function or the promiscuous nature of compounds binding to specific types of proteins. We can infer the relationships between targets and their drugs in order to characterize the molecular properties of compounds with specific ADMET properties and adverse effects. For a new compound or target, IDAAPM can be exploited in regard to probable mode of administration and status (Fig. 2a), target class and drug area (Fig. 2b) as well as adverse effect and the drug area of current drugs in the database (Fig. 3a).

IDAAPM enables researchers to perform data analysis and data mining from safe drugs data and to identify the space of similar compounds and similar targets. This is particularly important for prediction of possible ADMET properties or adverse effects of new drug molecules. Furthermore, this will allow the prospect of *in silico* approaches to be used for drug repurposing through the integration of knowledge from IDAAPM.

Three examples of usage are presented. In the first example, IDAAPM usage coupled with KNIME capabilities is presented in Fig. 4c, d. First, on Fig. 4c, for a set of compounds selected from IDAAPM, a distance matrix can be calculated and used to characterize the chemical space (heat map and 2D/3D scatter plot) as well as cluster analysis (hierarchical clustering) of the compounds selected by querying IDAAPM (Fig. 4c). Then, on Fig. 4d, a structure similarity towards IDAAPM compounds is exemplified to identify the most similar or dissimilar compounds using for example Tanimoto similarity coefficient (Fig. 4d). As a result, a 2D/3D structural similarity matrix is obtained by computing Tanimoto distance values for all pairs of the compounds, included as a single column containing distance vector values.

**Fig. 5:**
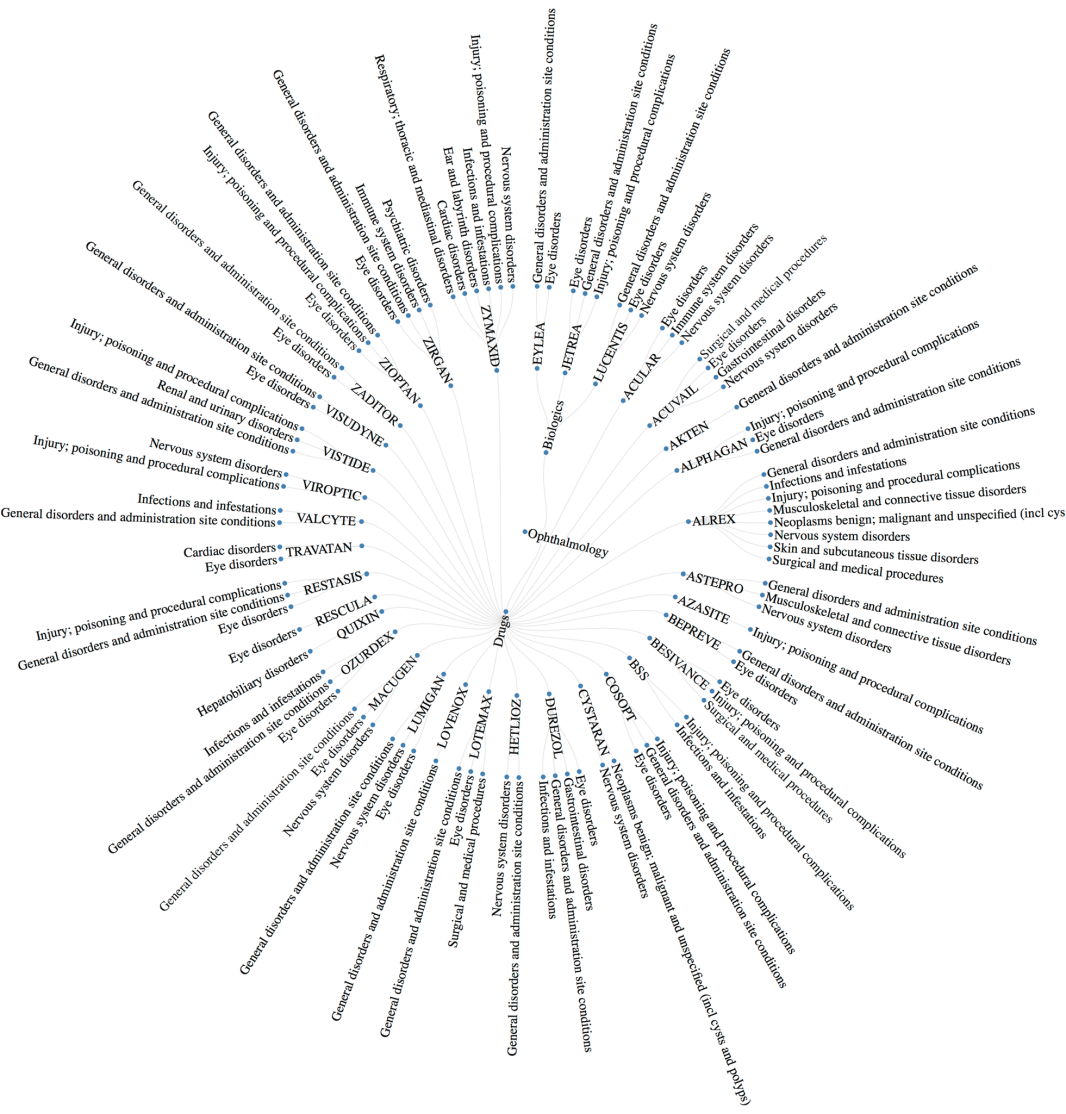
Ocular pharmaceutics adverse effect network with relative frequency >0.1. Adverse effects are reported using the system organ class of the medical dictionary for regulatory activities

In the second example, data mining on IDAAPM is demonstrated in the context of ocular pharmaceutics. Firstly, adverse effects are mined to identify the most frequent adverse effects for ocular drugs and their relative frequency (see Fig. 5). This can be combined with, for example ocular adverse effects for systemic drugs (see Fig. 3b). The data can be analyzed to connect drugs, drug targets and their adverse effects, delivering a powerful data mining approach for example to better understand polypharmacology or to predict ocular side effects. This is particularly important for prediction of possible adverse effects of new drug molecules. Furthermore, this will allow the prospect of in silico approaches being utilized for drug repurposing through the integration of knowledge from IDAAPM.

A third example of usage is provided by a workflow (in Fig. 4e) used to build predictive classification models inside KNIME from bioactivity data, ADMET, adverse effects, or molecular descriptors selected from IDAAPM is demonstrated. Data are uploaded from .sdf files exported from a search result made on IDAAPM. As seen in this example, the data are first normalized, then separated into training and test sets. This is followed by model construction, either using K-nearest neighbors or the support vector machine method. The stability of the trained model can be verified through cross validation during the process and its predictive ability can be estimated using the external test set. The scorer node reports a confusion matrix and the related accuracy statistic of generated models.

## Conclusion

IDAAPM offers structured and manually curated data on approved drugs, physicochemical properties, ADMET and adverse effects as well as related target information. It provides measured bioactivities, also structural information, which is essential in predictive modeling. IDAAPM combines detailed information on approved drugs in a single platform integrated with KNIME. The KNIME workflow has been developed to allow for easy access to the safe compound information and further provide the researcher with more opportunity to develop their own custom workflow, depending on their needs. The availability of the data in an easily accessible form will allow researchers in the field to easily use this data as well as allow easy integration with other programs and services. It will provide data from available safe drugs and knowledge for drug discovery and development.

Further data curation, comprehensive data coverage and improvements are planned for subsequent IDAAPM releases, as well as inclusion of additional predictive modeling KNIME workflows. Moreover, we aim to include all compounds in advanced clinical trials, as this is highly relevant in the context of drug discovery. Even though the first release of IDAAPM includes experimental and calculated physico-chemical properties, we aim to include additional molecular properties.

## Availability

IDAAPM full database and KNIME workflows are freely accessible at http://idaapm.helsinki.fi.

## Additional files

10.1186/s13321-016-0141-7 Flowchart of the overall project. Simplified representation of different steps needed for IDAAPM construction from data sources, contents, processes to the integration with KNIME workflow environment.
10.1186/s13321-016-0141-7 Figure of the full entity relationship model of IDAAPM.
10.1186/s13321-016-0141-7 IDAAPM entity relation schema model documentation. This documentation detailed the format of data and table in IDAAPM and how they are linked.
10.1186/s13321-016-0141-7 SQL script to store FDA Approved drug (Drug@FDA) to IDAAPM.
10.1186/s13321-016-0141-7 R script to extract the adverse effects data from FAERS xml file.
10.1186/s13321-016-0141-7 SQL script to store FAERS adverse effect from CSV file to IDAAPM.
10.1186/s13321-016-0141-7 R script to extract data from DrugBank XML file to a CSV file.
10.1186/s13321-016-0141-7 SQL script to store data from CSV DrugBank file (ADMET, molecular descriptors) to IDAAPM.
10.1186/s13321-016-0141-7 SQL script to extract and store data (target and binding affinity) from BindingDB TSV file to IDAAPM.
10.1186/s13321-016-0141-7 Chart plot of IDAAPM content per sources. Summary of different types of data and corresponding sources present in IDAAPM as well as their relative proportion in comparison to the overall Database.
10.1186/s13321-016-0141-7 IDAAPM main graphical user interface (A) with example of query (B) and search results (C).
10.1186/s13321-016-0141-7 Main KNIME node (FDA Approved Dugs products node on Fig. 4, panel A) to access IDAAPM. This window helps to configure the query to be sent to IDAAPM with several options to filter the data.

## References

[1] Bunnage ME (2011). Getting pharmaceutical R&D back on target. Nat Chem Biol.

[2] Hay M, Thomas DW, Craighead JL, Economides C, Rosenthal J (2014). Clinical development success rates for investigational drugs. Nat Biotechnol.

[3] Kola I, Landis J (2004). Can the pharmaceutical industry reduce attrition rates?. Nat Rev Drug Discov.

[4] Dearden JC (2007). In silico prediction of ADMET properties: how far have we come?. Expert Opin Drug Metab Toxicol.

[5] Gleeson MP, Hersey A, Hannongbua S (2011). In-silico ADME models: a general assessment of their utility in drug discovery applications. Curr Top Med Chem.

[6] Gleeson MP, Modi S, Bender A, Robinson RL, Kirchmair J, Promkatkaew M, Hannongbua S, Glen RC (2012). The challenges involved in modeling toxicity data in silico: a review. Curr Pharm Des.

[7] Moroy G, Martiny VY, Vayer P, Villoutreix BO, Miteva MA (2012). Toward in silico structure-based ADMET prediction in drug discovery. Drug Discov Today.

[8] Raunio H (2011). In silico toxicology—non-testing methods. Front Pharmacol.

[9] Gleeson MP (2008). Generation of a set of simple, interpretable ADMET rules of thumb. J Med Chem.

[10] Leeson PD, Springthorpe B (2007). The influence of drug-like concepts on decision-making in medicinal chemistry. Nat Rev Drug Discov.

[11] Lipinski CA (2000). Drug-like properties and the causes of poor solubility and poor permeability. J Pharmacol Toxicol Methods.

[12] Price DA, Blagg J, Jones L, Greene N, Wager T (2009). Physicochemical drug properties associated with in vivo toxicological outcomes: a review. Expert Opin Drug Metab Toxicol.

[13] Hartung T, Hoffmann S (2009). Food for thought … on in silico methods in toxicology. Altex.

[14] Hou T (2015). Editorial. In silico ADMET predictions in pharmaceutical research. Adv Drug Deliv Rev.

[15] Modi S, Li J, Malcomber S, Moore C, Scott A, White A, Carmichael P (2012). Integrated in silico approaches for the prediction of Ames test mutagenicity. J Comput Aided Mol Des.

[16] Law V, Knox C, Djoumbou Y, Jewison T, Guo AC, Liu Y, Maciejewski A, Arndt D, Wilson M, Neveu V (2014). DrugBank 4.0: shedding new light on drug metabolism. Nucleic Acids Res.

[17] Gaulton A, Bellis LJ, Bento AP, Chambers J, Davies M, Hersey A, Light Y, McGlinchey S, Michalovich D, Al-Lazikani B (2012). ChEMBL: a large-scale bioactivity database for drug discovery. Nucleic Acids Res.

[18] Gilson MK, Liu T, Baitaluk M, Nicola G, Hwang L, Chong J (2016). BindingDB in 2015: a public database for medicinal chemistry, computational chemistry and systems pharmacology. Nucleic Acids Res.

[19] Kim S, Thiessen PA, Bolton EE, Chen J, Fu G, Gindulyte A, Han L, He J, He S, Shoemaker BA (2016). PubChem substance and compound databases. Nucleic Acids Res.

[20] Berman HM, Westbrook J, Feng Z, Gilliland G, Bhat TN, Weissig H, Shindyalov IN, Bourne PE (2000). The protein data bank. Nucleic Acids Res.

[21] Wang RX, Fang XL, Lu YP, Wang SM (2004). The PDBbind database: collection of binding affinities for protein-ligand complexes with known three-dimensional structures. J Med Chem.

[22] Southan C, Sharman JL, Benson HE, Faccenda E, Pawson AJ, Alexander SP, Buneman OP, Davenport AP, McGrath JC, Peters JA (2016). The IUPHAR/BPS Guide to PHARMACOLOGY in 2016: towards curated quantitative interactions between 1300 protein targets and 6000 ligands. Nucleic Acids Res.

[23] Yang H, Qin C, Li YH, Tao L, Zhou J, Yu CY, Xu F, Chen Z, Zhu F, Chen YZ (2016). Therapeutic target database update 2016: enriched resource for bench to clinical drug target and targeted pathway information. Nucleic Acids Res.

[24] Miller MA, Hazard GF, Hudson VW, Hilt C, Fang J, Mayer D, Callahan L (2003). ChemIDplus: a free, web-based portal to a variety of compound-based information. Abstr Pap Am Chem Soc.

[25] U.S. Food and Drug Administration Drugs@FDA. http://www.accessdata.fda.gov/scripts/cder/drugsatfda/

[26] Ahmad SR, Goetsch RA, Marks NS, Strom BL (2005). Spontaneous reporting in the United States. Pharmacoepidemiology.

[27] Harpaz R, Haerian K, Chase HS, Friedman C (2010). Statistical mining of potential drug interaction adverse effects in FDA’s spontaneous reporting system. AMIA Annu Symp Proc.

[28] Morrissey KM, Wen CC, Johns SJ, Zhang L, Huang SM, Giacomini KM (2012). The UCSF-FDA transportal: a public drug transporter database. Clin Pharmacol Ther.

[29] Sedykh A, Fourches D, Duan J, Hucke O, Garneau M, Zhu H, Bonneau P, Tropsha A (2013). Human intestinal transporter database: QSAR modeling and virtual profiling of drug uptake, efflux and interactions. Pharm Res.

[30] Moda TL, Torres LG, Carrara AE, Andricopulo AD (2008). PK/DB: database for pharmacokinetic properties and predictive in silico ADME models. Bioinformatics.

[31] Kuhn M, Campillos M, Letunic I, Jensen LJ, Bork P (2010) A side effect resource to capture phenotypic effects of drugs. Mol Syst Biol 6. doi:10.1038/msb.2009.98 10.1038/msb.2009.98 PMC2824526 20087340

[32] Cheng FX, Li WH, Wang XC, Zhou YD, Wu ZR, Shen J, Tang Y (2013). Adverse drug events: database construction and in silico prediction. J Chem Inf Model.

[33] Berthold MR, Cebron N, Dill F, Di Fatta G, Gabriel TR, Georg F, Meinl T, Ohl P, Sieb C, Wiswedel B (2006) Knime: The konstanz information miner. In: 4th international industrial simulation conference 2006, pp 58–61

[34] Giannangelo K (2006) Principles to guide maintenance of classifications. In: Reichert A, Mihalas G, Stoicu-Tivadar L et al (eds) Proceedings of the EFMI special topic conference. Integrating biomedical information: from E-cell to E-patient, Timisoara, Romania, 6–8 April 2006. AKA-Verlag, Berlin, pp 293–297

