# Supplementary figures and images for: IDAAPM: integrated database of ADMET and adverse effects of predictive modeling based on FDA approved drug data
Source: J Cheminform. 2016 Jun 14;8:33. doi: 10.1186/s13321-016-0141-7 (PMC4906584; doi:10.1186/s13321-016-0141-7)

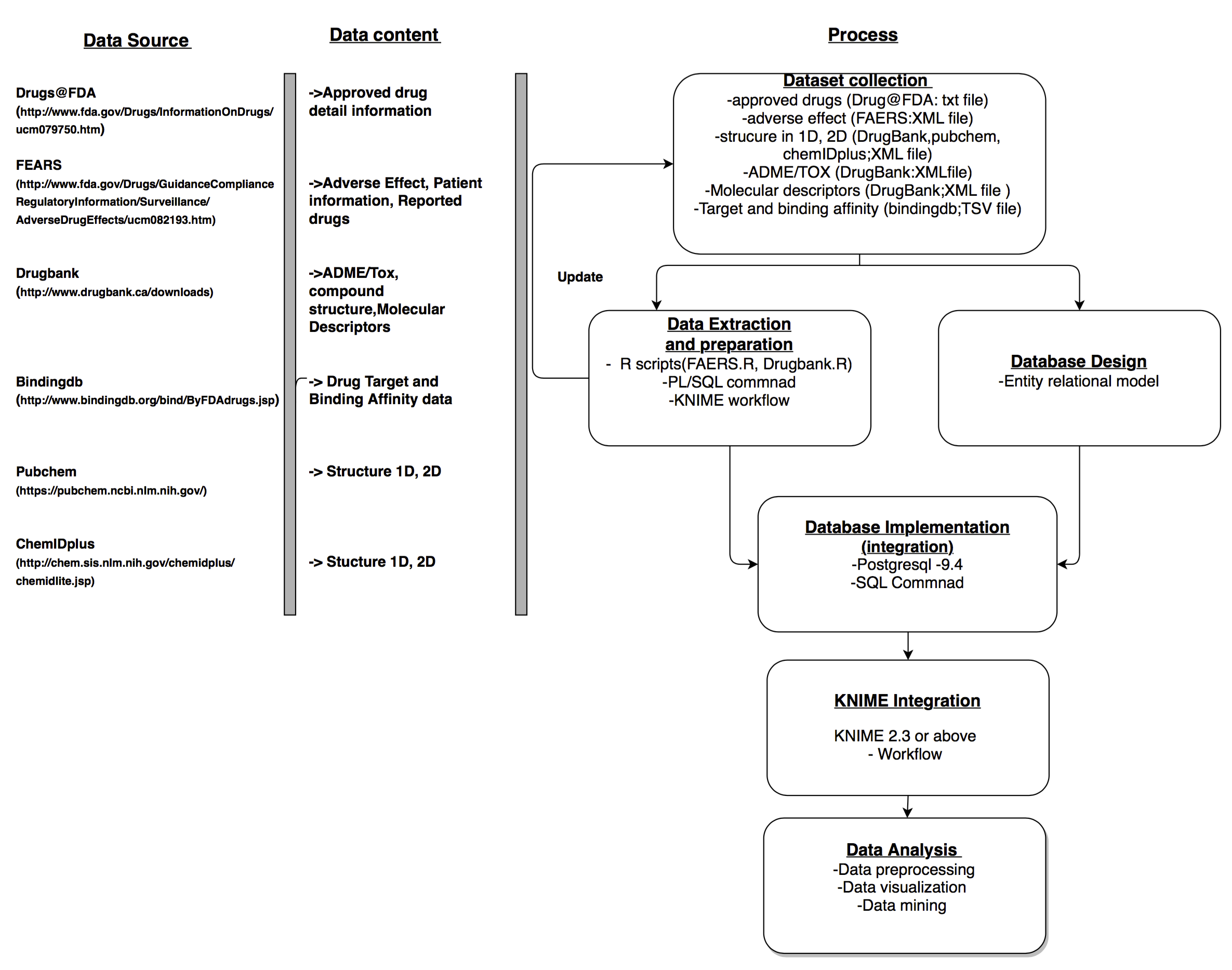

Supplement: Supplementary file 1 — 10.1186/s13321-016-0141-7 Flowchart of the overall project. Simplified representation of different steps needed for IDAAPM construction from data sources, contents, processes to the integration with KNIME workflow environment. [file 13321_2016_141_MOESM1_ESM.tiff]

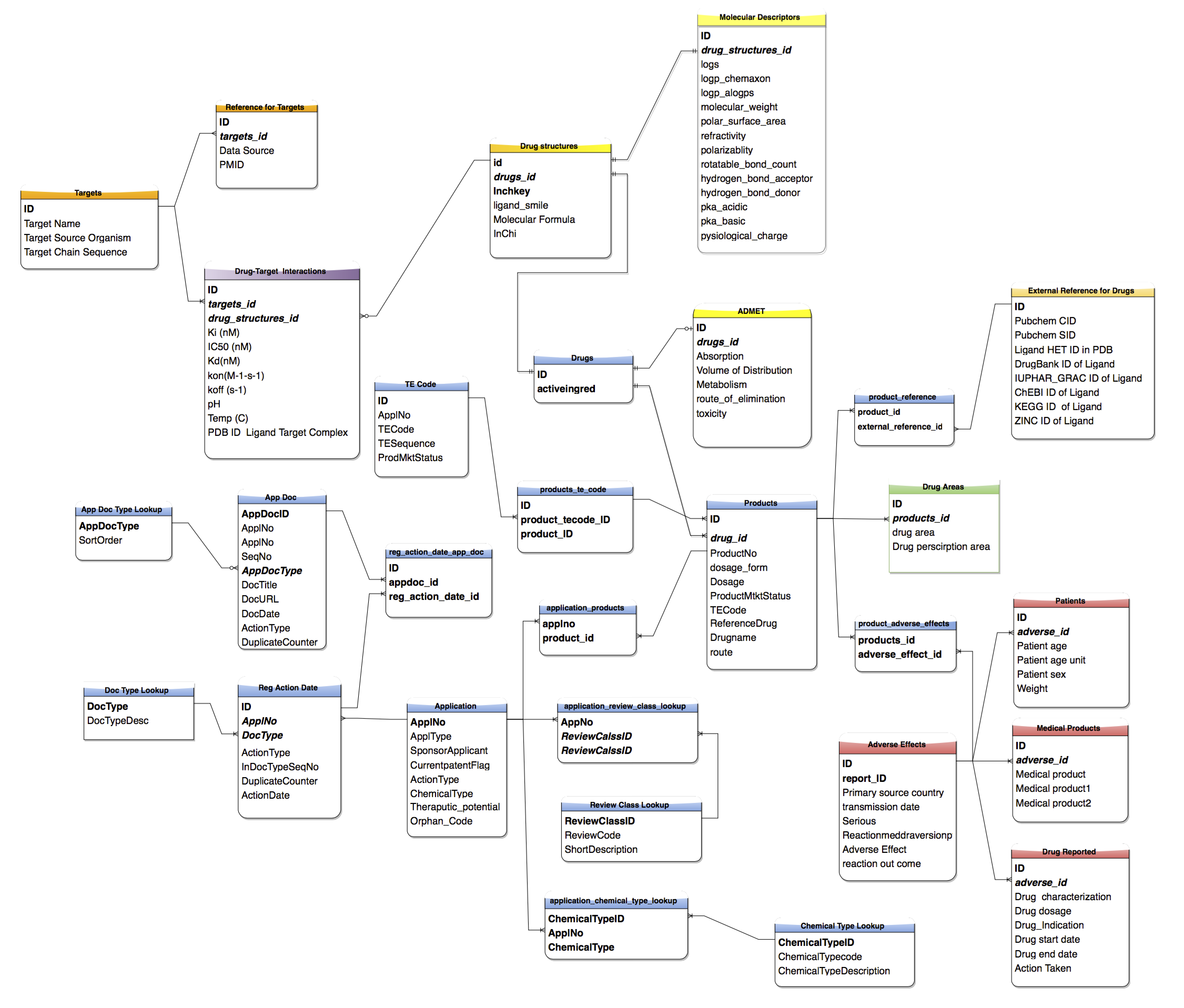

Supplement: Supplementary file 2 — 10.1186/s13321-016-0141-7 Figure of the full entity relationship model of IDAAPM. [file 13321_2016_141_MOESM2_ESM.tiff]

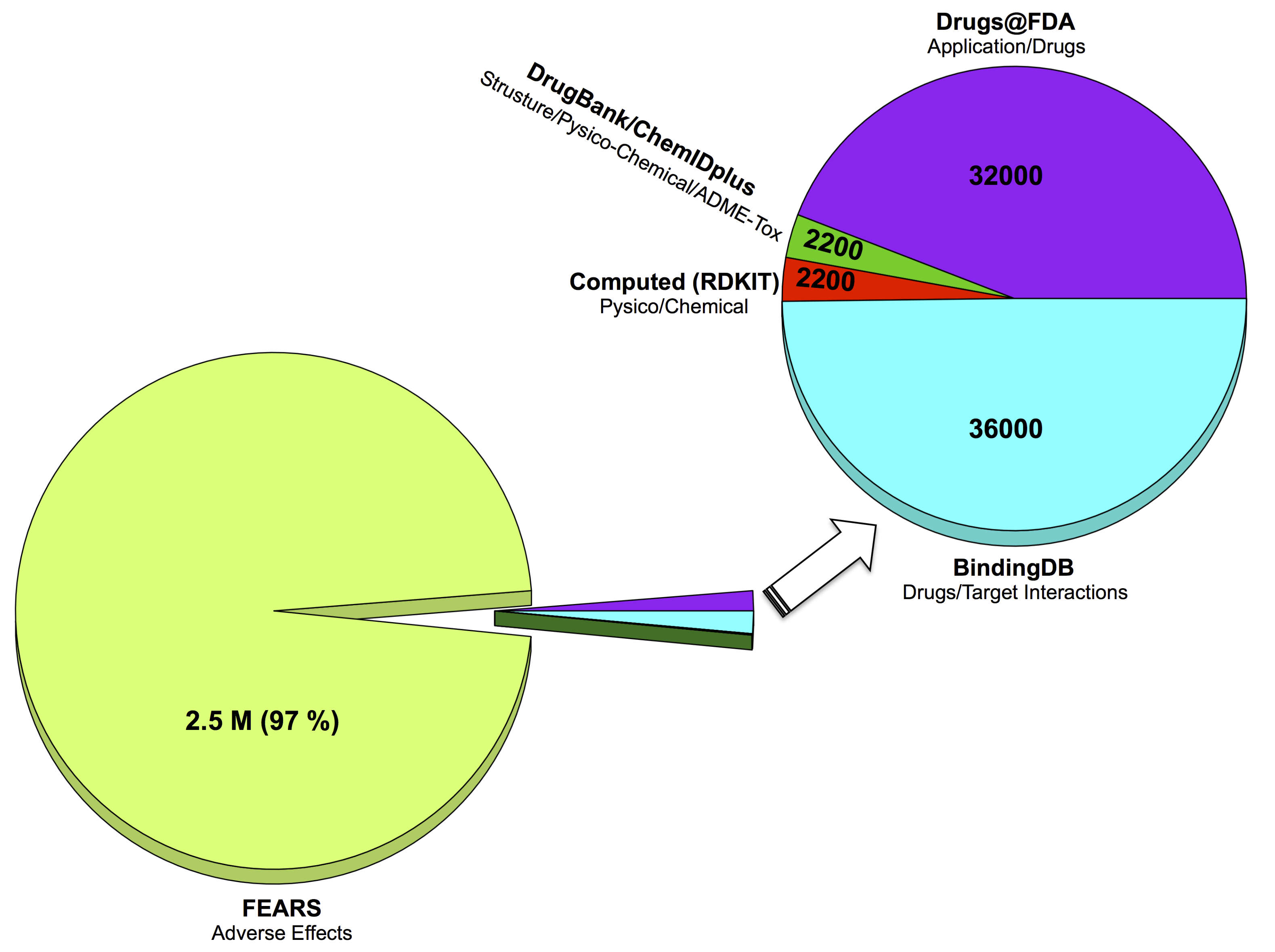

Supplement: Supplementary file 10 — 10.1186/s13321-016-0141-7 Chart plot of IDAAPM content per sources. Summary of different types of data and corresponding sources present in IDAAPM as well as their relative proportion in comparison to the overall Database. [file 13321_2016_141_MOESM10_ESM.tiff]

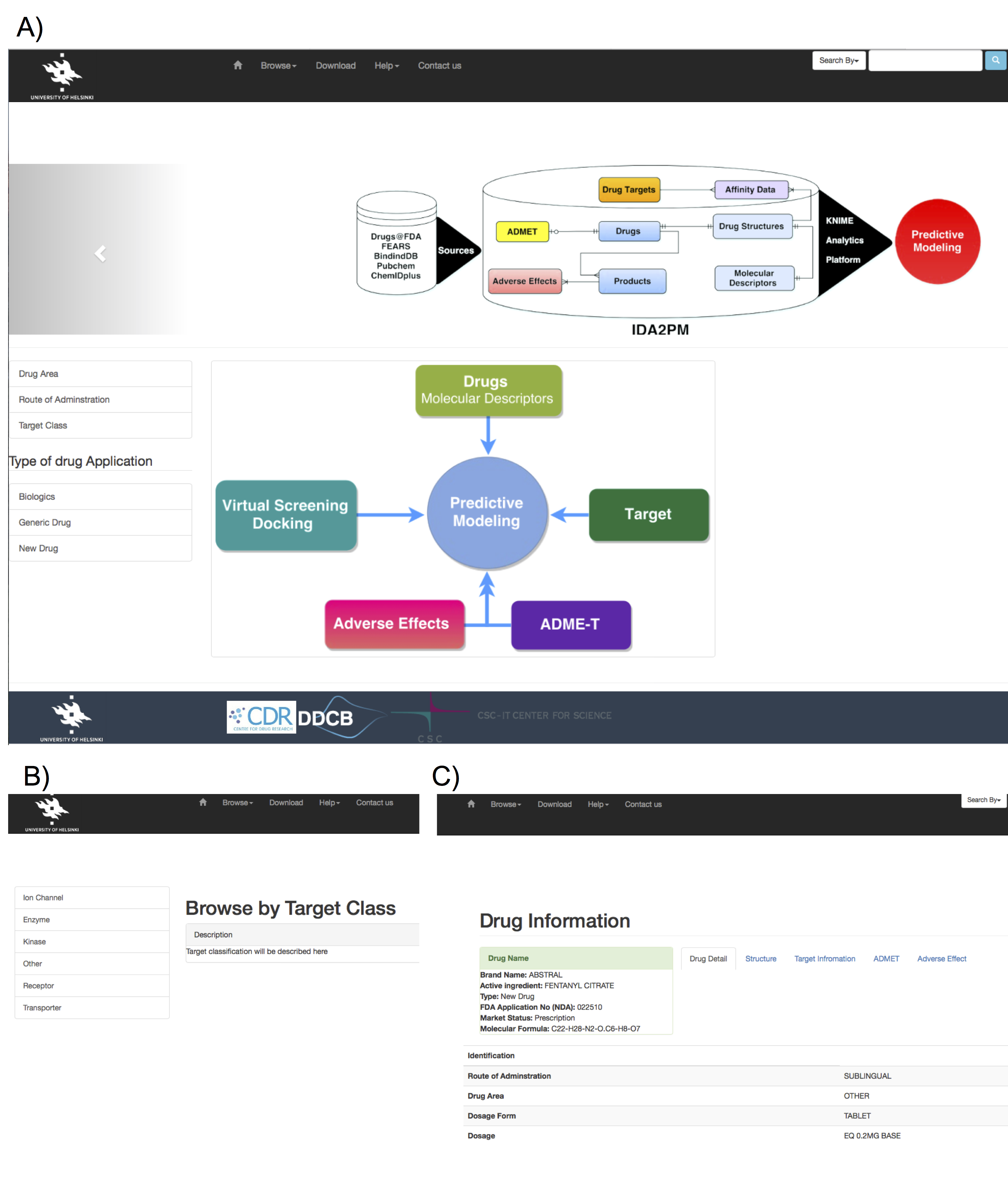

Supplement: Supplementary file 11 — 10.1186/s13321-016-0141-7 IDAAPM main graphical user interface (A) with example of query (B) and search results (C). [file 13321_2016_141_MOESM11_ESM.tiff]

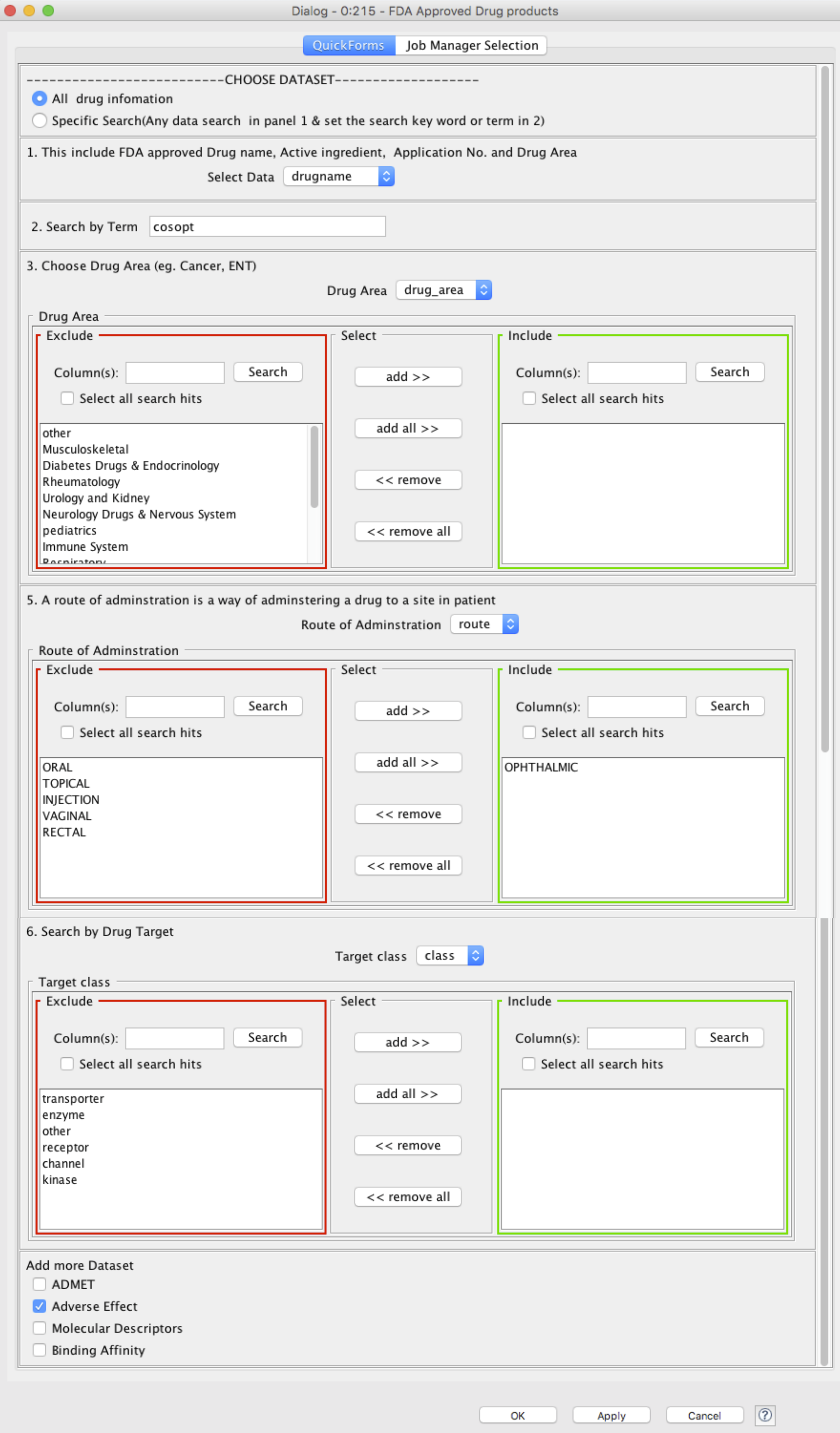

Supplement: Supplementary file 12 — 10.1186/s13321-016-0141-7 Main KNIME node (FDA Approved Dugs products node on Fig. 4, panel A) to access IDAAPM. This window helps to configure the query to be sent to IDAAPM with several options to filter the data. [file 13321_2016_141_MOESM12_ESM.tiff]
